# Flexible Adhesive in Composite-to-Brick Strengthening—Experimental and Numerical Study

**DOI:** 10.3390/polym10040356

**Published:** 2018-03-22

**Authors:** Arkadiusz Kwiecień, Piotr Krajewski, Łukasz Hojdys, Marcin Tekieli, Marek Słoński

**Affiliations:** 1 Institute of Structural Mechanics, Cracow University of Technology, 31-155 Cracow, Poland; arkadiusz.kwiecien@pk.edu.pl; 2 Institute of Building Materials and Structures, Cracow University of Technology, 31-155 Cracow, Poland; lukasz.hojdys@pk.edu.pl; 3 Institute for Computational Civil Engineering, Cracow University of Technology, 31-155 Cracow, Poland; marcin.tekieli@l5.pk.edu.pl (M.T.); marek.slonski@L5.pk.edu.pl (M.S.)

**Keywords:** strengthening, masonry, CFRP, CFRPU, SRP, SRPU, flexible adhesives, experimental tests, DIC measurements, shear bond tests, numerical analysis

## Abstract

This paper investigates composite-to-brick strengthening systems with flexible adhesive made of polyurethane (Carbon Fibre Reinforced Polyurethane (CFRPU) and Steel Reinforced Polyurethane (SRPU)) and epoxy resin (Carbon Fibre Reinforced Polymer (CFRP) and Steel Reinforced Polymer (SRP). The specimens were tested in a single lap shear test (SLST). LVDT displacement transducers (LVDT – Linear Variable Differential Transformer) and digital image correlation method (DIC) based measurement systems were used to measure displacements and strains. The obtained results were applied in a numerical analysis of the 3D model of the SLST specimen, with flexible adhesives modeled as a hyper-elastic model. The DIC and LVDT based systems demonstrated a good correlation. Experimental and numerical analysis confirmed that composite-to-brick strengthening systems with flexible adhesives are more effective on brittle substrates than stiff ones, as they are able to reduce stress concentrations and more evenly distribute stress along the entire bonded length, thus having a higher load carrying capacity.

## 1. Introduction

Composite strengthening of masonry structures is now one of the most popular methods for improving load capacity and ductility of existing masonries. The rapid increase of applications in practice of such systems is visible, particularly in Italy, where earthquakes severely damaged many structures over the last decades. The first composite strengthening applications were introduced, in practice, with the use of stiff epoxy resin adhesives. When maximum loads were reached, these adhesives caused serious damages to masonry substrates. In addition, these strengthening methods were found to be irreversible to and incompatible with masonry substrates, a factor that resulted in the heritage structure authorities’ disapproval of their application. Moreover, the effectiveness of such composites bonded on stiff epoxies was determined to be low, because of the short effective bonding length and the generated high stress concentrations, responsible for the fracture in a brittle substrate. This problem prompted the search for a new, more compatible and effective strengthening system that utilized mineral mortars or highly deformable polyurethanes.

Fibre Reinforced Polymers (FRP) have been investigated intensively and applied in practice for structural strengthening over the last decade [1,2,3,4,5]. The stiff and brittle adhesives, as epoxy resin [6,7,8] or mineral mortars [9,10,11,12,13,14], are applied in composite strengthening systems composed of various high strength fibers, bonded to concrete and masonry substrates. Composite reinforcement textiles, made of various fibers, are divided into groups according to the fiber and matrix/adhesive type. There are various known strengthening systems that are bonded using stiff epoxy matrices (FRP—Fibre Reinforced Polymers or SRP—Steel Reinforced Polymers) and stiff mineral matrices (FRCM—Fibre Reinforced Cementitious Mortar, TRM—Textile Reinforced Mortar, SRG—Steel Reinforced Grout). In such systems, peaks of stress concentration were observed [15] with low effective bonding lengths (inversely proportional to the stiffness of the adhesives), causing fracture damages to brittle substrates when the maximum fracture energy was reached. The real behavior of FRP systems, strengthening full scale masonry specimens, was presented in [16,17], where stiff epoxy adhesives fully utilized the fracture energy of the brittle substrate. This research clearly presented that a longer bonding length of a stiffly glued composite does not increase the bond strength (because of the limited fracture energy) until additional strengthening systems are applied (the vacuum method and the anchor spike method). As a new approach, this studied proposed highly deformable polyurethane adhesives, which allow the bond strength to increase significantly by increasing the effective bonding length, even without fully utilizing the fracture energy of a masonry substrate [18].

In the last few years, the applications of flexible polyurethane adhesives FRPU/SRPU (Fiber/Steel Reinforced Polyurethanes) were tested on masonry substrates [18,19,20,21,22,23,24]. The effectiveness of composite strengthening, bonded on flexible adhesives to concrete substrates was also demonstrated [25,26]. This confirmed that highly deformable polyurethane adhesives introduce additional ductility in a strengthened structure, without the loss of global strength and stiffness in comparison to structures strengthened with composites bonded on stiff epoxy adhesives. The results showed an increase in maximum load, ultimate slip, and ultimate work for the composite systems with flexible adhesives, in comparison to the same parameters for systems with stiff adhesives.

The application of polymeric resins in the strengthening of masonry structures is not recommended by authorities, because of prior negative experiences with epoxy resin adhesives and injections. The observed problems were caused by mechanical, thermal, and chemical incompatibility when stiff and high strength epoxies caused damages to weak masonry substrates, especially heritage ones [27]. To avoid these significant disadvantages, a new family of organic materials, based on highly deformable polyurethanes of low stiffness, was developed. These materials, presented in this paper, fulfill the requirements of the authorities. They are mechanically compatible with masonries, do not generate dangerous stresses during thermal expansion, are chemically neutral, and are reversible, confirmed in various tests [19,28,29], including durability tests under authority supervision [22].

The flexibility of an adhesive layer is the key issue when considering the reduction of stress concentration and stress redistribution, and it can be effectively observed using a digital image correlation method (DIC) [30]. This novel method of measurement has already been successfully employed in mechanical testing on composites. DIC has been used in the investigation of the FRP-to-concrete [31,32,33] and FRP-to-masonry [32,34,35], FRCM composites [36] bond behavior, and large-scale testing [37,38]. The potential application of DIC to all steps of the composite materials with both polymer and mortar matrices characterization, to be used as externally bonded reinforcements, has not yet been deeply investigated and is discussed in detail in [30].

The motivation and objective of this study was to confirm that those less stiff polyurethane adhesives, rather than the classically used, stiff epoxy resins, allow for an increase in the effective bonding length of composite-to-masonry strengthening, and thus, the bond strength of a system. By increasing in this way, the load capacity was expected to be proof of the flexible adhesive’s ability to increase the effectiveness of composite-to-brick strengthening systems, in comparison to stiff ones. To confirm this phenomenon, experimental tests were carried out using two measuring systems: traditional and DIC based CivEng-Vision software ver. 1.2 [30] (developed at the Cracow University of Technology (CUT)). A 3D numerical approach was also applied using linear and nonlinear numerical models of the tested materials. Polyurethane flexible adhesives have a hyper-elastic behavior; thus, the appropriate Mooney-Rivlin model was adopted for numerical analysis, which was applied previously, in the analysis of the CFRPU (Carbon Fibre Reinforced Polyurethane) [18] and SRPU (Steel Reinforced Polyurethane) [22] systems. Specimens of composite-to-brick strengthening systems were tested in a single lap shear test (SLST) at CUT with a flexible adhesive made of polyurethane (CFRPU and SRPU) and epoxy resin (Carbon Fibre Reinforced Polymer (CFRP) and Steel Reinforced Polymer (SRP)). The SLST set-ups used at CUT were similar to those recommended by RILEM TC223MSC and RILEM TC250CSM [6,13,23,39].

The use of various testing and analysis methods allowed for a reliable comparison of behavior and effect of the stiff and flexible adhesive’s application in bonding of composites to a brittle substrate, as well as a presentation of differences in strain distribution along the bond length. The load capacity and ductility behavior were compared for various strengthening systems through the presentation of load-slip curves, whereas a lack of the full utilization of the tested substrate fracture energy was validated by the observed failure modes. On the other hand, the load capacity vs. adhesive stiffness clearly presents how the effective bond strength changes with the modification of the adhesive elastic modulus. In addition, the strain distribution, determined by various methods (strain gauges, DIC, and numerical calculations), illustrated effective bonding length and the presence of strain (and thus stress) concentrations when composites were bonded using stiff and flexible adhesives.

## 2. Laboratory Tests

The experiment was divided into two parts. First, some preliminary tests on the carbon textile ( CFRP and CFRPU) and ultra high steel textile (SRP and SRPU) based strengthening systems were carried out, in order to understand the influence of the stiffness of the matrix on the composite-to-brick bond behavior. In these tests, the set-up used in Round Robin Tests (RRT) of RILEM TC223MSC [6,23] was applied, with bond length of 200 mm. In the second phase, the other two strengthening systems were analyzed: CFRP laminates and ultra-high strength steel textile bonded to brick substrates. In these tests, the set-up used in Round Robin Tests (RRT) of RILEM TC250CSM [13,39] was applied. In all second phase tests, three different adhesives were adopted in order to determine their bond properties. An optical measurement system was used to measure the displacement and strain fields of the samples throughout the tests.

### 2.1. Materials and Specimen Preparation

**Figure 1:**
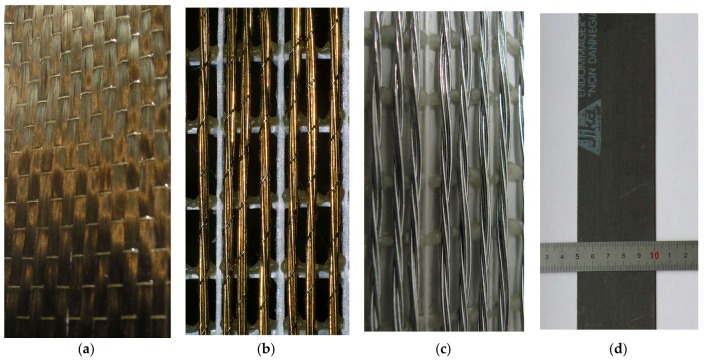
Textiles and laminates adopted in research: (**a**) carbon textile; (**b**) steel textile S1—detail; (**c**) steel textile S2—detail; (**d**) CFRP strip.

All of the tests were carried out on a single type of substrate, namely single clay bricks. The bricks RossoVivo A6R55W were of the following dimensions: 250 mm long, 120 mm wide, and 55 mm thick (the same as those used in RILEM TC250 and RILEM TC223 experiments [6,13,39]). The strengthening systems were made from a combination of carbon textile, two various steel textiles or CFRP laminates with four various adhesives: epoxy based (Sikadur 330 and Sikadur 30) [23], polyurethane (PS) [15], and polyurethane (PT) [40]. These were applied following unidirectional strengthening composites (50 mm-wide): carbon (FIDCARBON UNI 320 HT240) textile and steel (FIDSTEEL 3X2-B 12-12-500 [6,41] and Kerakoll GeoSteel G2000) [42] textiles (Figure 1). Their weights were 320 g/m^2^, 1800 g/m^2^ and 2000 g/m^2^, respectively. Additionally, pultruded CFRP laminate, also 50 mm-wide (a half of Sika CarboDur S1012 laminate) and 1.2 mm-thick, was used in the tests [43] (Figure 1d). The mechanical properties of epoxy and polyurethane adhesives were obtained from the producer’s data sheets. The main mechanical properties of the adopted materials are given in Table 1.

**Figure 2:**
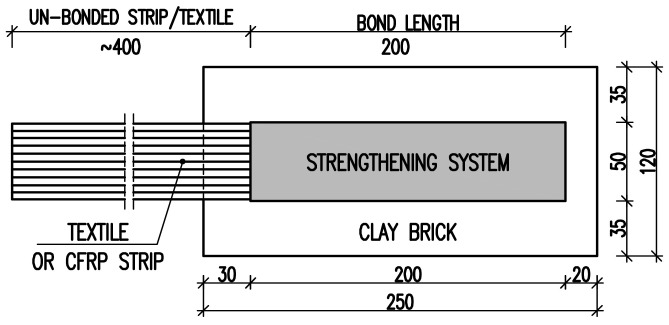
Specimen geometry, dimensions in mm.

The specimens for the single-lap shear tests were prepared by bonding a single, 50 mm-wide strip of textile or CFRP laminate along the centerline of the bed face of a single brick. As the steel fiber textile consists of steel micro-cords fixed to a fiberglass micromesh, two sides of this textile were considered: side M (glass mesh side) and side S (steel cords side). The composite was located at a distance of 20 mm from the upper header face (unloaded side) and 35 mm from stretcher face. The bond length was equal to 200 mm. The length of a free, un-bonded strip was about 400 mm. The geometry of the specimens is presented in Figure 2.

**Figure 3:**
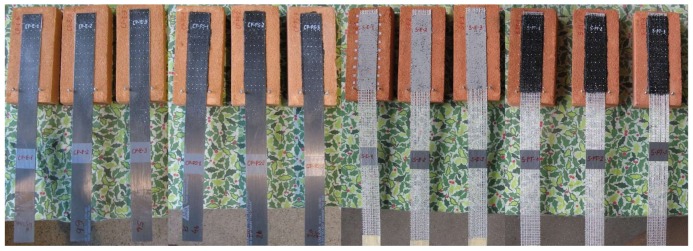
Manufactured single-lap shear test specimens—from left to right (3 in each group): CP-E, CP-PS, S2/S-E and S2/S-PT.

In total, ten various types of specimens were prepared and tested. The notations used in the research and short descriptions of tested specimens are presented in Table 2. The name of the specimen consists of: type of reinforcement (C—carbon fiber textile, S1—steel fiber textile S1, S2—steel fiber textile S2, CP—pultruded CFRP laminate); type of adhesive/matrix (E—epoxy-based, PS—polyurethane PS, PT—polyurethane PT). Additionally, labels “/M” and “/S” were used for textiles with glass mesh side and steel cords side placed towards the substrate, respectively. They are also grouped according to the test set-ups used in the first (TS-1) and the second phase (TS-2), as is described later in this paper. All specimens were manufactured according to RILEM TC223 and RILEM TC250 requirements [6,9,10,11,12,13]. Several of the tested specimens are presented in Figure 3.

### 2.2. Test Set-Ups and Experimental Procedure

In the tests, a single push-pull configuration for the test set-ups was used. The reinforcement of the composite is bonded only on one side and is pulled while the brick is restrained and partially compressed [6,9,10,11,12,13].

**Figure 4:**
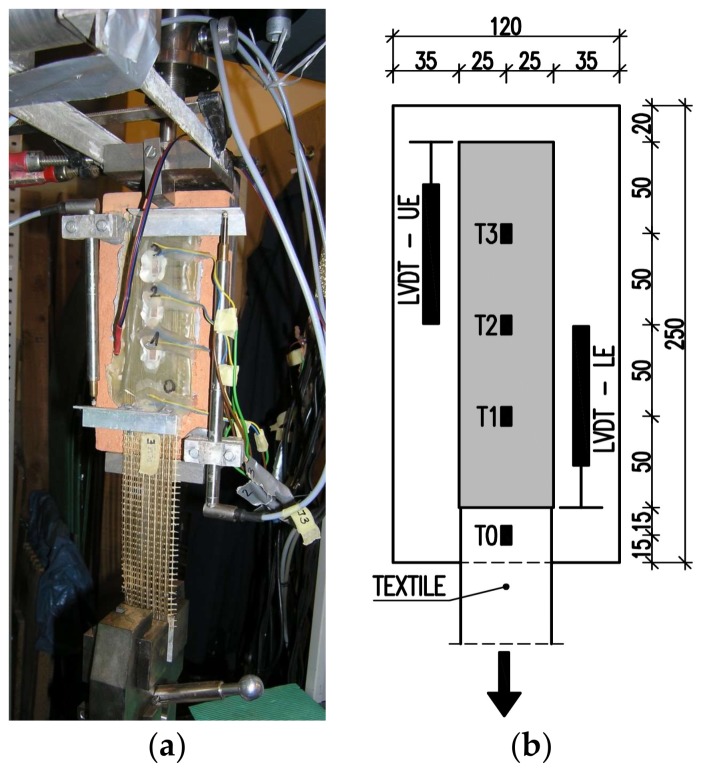
(**a**) Test set-up TS-1 with supporting C-shaped steel frame; (**b**) scheme with location of strain gauges and LVDTs, dimensions in mm.

The specimens used in the first phase of the experiments (TS-1) were placed in a balanced C-shaped steel frame with a brick rotation blockade (Figure 4a). The frame was placed in the universal testing machine (Zwick 1455 20 kN; Zwick Roell, Ulm, Germany) and fixed at the top using a ball hinge. The free end of the reinforcement was clamped in wedge grips and loaded. Aluminum tabs were used at the free end of textile/strip to avoid slippage at the clamps and to guarantee a homogeneous stress distribution. The load and the slippage between the substrate and strengthening were measured during the tests. One LVDT (HBM WA/20MM-L) for slippage control was used at the loaded end and the second one (HBM WA/50MM-L) was used at the unloaded end (Figure 4b). In this phase of tests (TS-1), four strain gauges were glued along the centerline of the reinforcement to control strain changes (Figure 4b).

**Figure 5:**
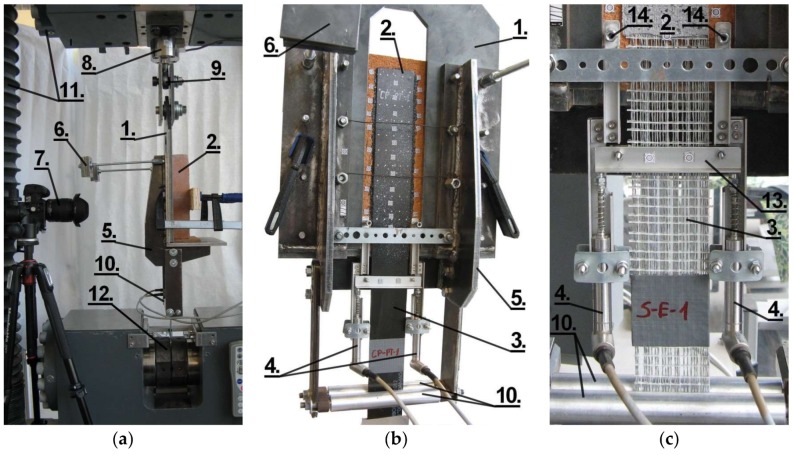
Test set-up TS-2: (**a**) side view; (**b**) front view; (**c**) detail—LVDTs. 1: Supporting L-shaped steel frame; 2. Tested specimen; 3. Loaded end of CFRP/textile; 4. LVDTs: L1—left one, L2—right one; 5. Stiffening rib; 6. Counterweight; 7. Camera for digital image correlation method (DIC); 8. Force transducer; 9. Ball-and-socket joint; 10. Aluminum guide rollers; 11. Testing frame; 12. Testing machine hydraulic clamp; 13. Aluminum section attached to loaded end of CFRP/textile; 14. LVDT holder-to-brick fastening.

The specimens used in the second phase (TS-2) were placed in an L-shaped steel frame, stiffened with two steel ribs to avoid rotations and distortions of the tested specimens (Figure 5). The supporting L-shaped frame was connected to the testing frame of a universal testing machine (Zwick Z1600 Zwick Roell, Ulm, Germany) by ball-and-socket joint. The free end of the textile/strip was passed between two aluminum guide rollers to ensure that the strengthening remains parallel to the surface of the substrate during the entirety of the test. In order to avoid slippage at the hydraulic clamps and to guarantee homogeneous stress distribution, two aluminum tabs were glued to the free end of textile/strip. In this case, two LVDTs (HBM WA/10MM-L), placed in the plane of textile/strip, were used to measure the slippage between the brick and composite reinforcement (Figure 5c). Additionally, the DIC method was used to measure displacements and strains. In order to provide an accurate load measurement, an additional force transducer HBM U2B 20 kN was used. The details of the test set-up TS-2 are presented in Figure 5.

All tests were performed under displacement control, with the displacement rate equal to 0.3 mm/min. The loads and displacements were acquired with an acquisition rate of 5 Hz.

The test setups were described and discussed in details in [6,18] and in [9,10,11,12,13] for TS-1 and TS-2 respectively.

### 2.3. DIC

The digital image correlation method (DIC) has been widely described and used for tests on materials in mechanical engineering. The DIC has also already been successfully used in experiments on composites, devoted to various fibers, matrices, and substrates on small specimens. The optical measurement system (CivEng Vision) used for the analysis of tested specimens has been developed by Tekieli and Słoński at CUT [45,46,47].

**Figure 6:**
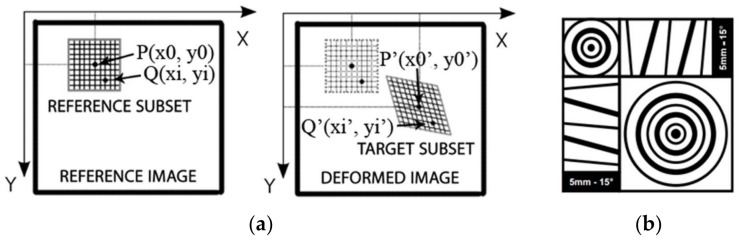
(**a**) principle of the Digital Image Correlation method; (**b**) the artificial marker with indication of real dimensions—[30].

The DIC method is based on the correlation of the digital images obtained during the test, which are treated as a two-dimensional matrix consisting of pixels. Each pair of the pictures taken before and after partial deformation are correlated, and the points of the grid based on the specified image subsets (Figure 6a) are matched and identified as that associated to the highest value of the correlation coefficient. This coefficient is calculated between the reference subset “f” and the target subset “g”, whose dimensions are equal and are *M* × *N* pixels using the zero mean normalized cross correlation method, as described by the formula below:

(1) $$CC^{ZMN}=\frac{\sum_{i=1}^{M}\sum_{j=1}^{N}\left(\left(f\left(i,j\right)-\mu_{f}\right)\times \left(g\left(i,j\right)-\mu_{g}\right)\right)}{\sqrt{\sum_{i=1}^{M}\sum_{j=1}^{N}{\left(f\left(i,j\right)-\mu_{f}\right)}^{2}}\times \sqrt{\sum_{i=1}^{M}\sum_{j=1}^{N}{\left(g\left(i,j\right)-\mu_{g}\right)}^{2}}}$$

where *μ_f_* is the luminosity of the reference subset and *μ_g_* is the luminosity of target subset.

The displacements of the image subsets obtained using DIC are originally computed in pixels and then converted into millimeters. To achieve this, the transition factor is used and calculated earlier using a calibration pattern.

In most cases, a speckle pattern, consisting of contrasted dots randomly distributed over a background, needs to be created on the specimen surface. It is also possible to place some artificial markers (Figure 6b) on the specimen to influence the accuracy of the results in specific points. Both of these are presented on the tested specimens (Figure 5).

To increase the resolution of the optical measurement, a subpixel interpolation is made. Based on the integer values of the pixels’ intensity, sub values are computed using cubic spline interpolation. Therefore, it is possible to gain an optical measurement resolution level of one hundredth of a pixel.

### 2.4. SLST Results

**Figure 7:**
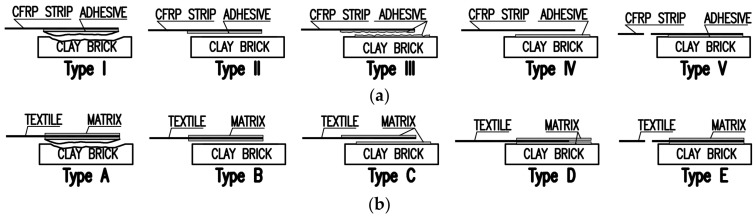
Possible failure modes for: (**a**) externally bonded CFRP strips; (**b**) textile wet lay-up systems on brick/masonry substrates.

The failure modes, obtained for specimens with textiles and CFRP plate, were specified and grouped separately, according to their differences. In the case of the CFRP ones, five characteristic failure modes were observed: type I—debonding of the plate with cohesive failure within the substrate, type II—detachment at the adhesive-to-substrate interface, type III—cohesive debonding within the adhesive, type IV—detachment at the plate-to-adhesive interface, type V—tensile failure of the plate (Figure 7a). The textile wet lay-up systems could also fail in five ways: type A—debonding of the composite with cohesive failure within the substrate, type B—detachment at the matrix-to-substrate interface, type C—cohesive failure in matrix/debonding at the textile-to-matrix interface, type D—textile slippage within the matrix, type E—tensile rupture of the textile (Figure 7b) [6,9,10,11,12,13].

The main failure mode, which was observed during the tests performed using the set-up TS-1 (specimens C-E, C-PS, S1/M-E and S1/M-PS), was cohesive failure within the substrate (type A) (see Figure 7a). In two cases (specimens C-PS-1 and C-PS-2) a non-standard failure mode occurred (due to shear failure of the brick), most likely caused by the configuration of the set-up TS-1 (introducing bending moment in a substrate) and some defects in the bricks [18]. This prompted the changing of the test set-up TS-1 to the test set-up TS-2 for further tests. In the first phase of the tests, the average failure loads were equal to 7.03 kN and 12.40 kN for carbon textile embedded in epoxy-based (C-E) and polyurethane PS matrix (C-PS), respectively. Specimens reinforced with steel cords S1 failed at a load of 6.78 kN for epoxy-based matrix (S1/M-E) and 12.02 kN for polyurethane ones (S1/M-PS).

**Figure 8:**
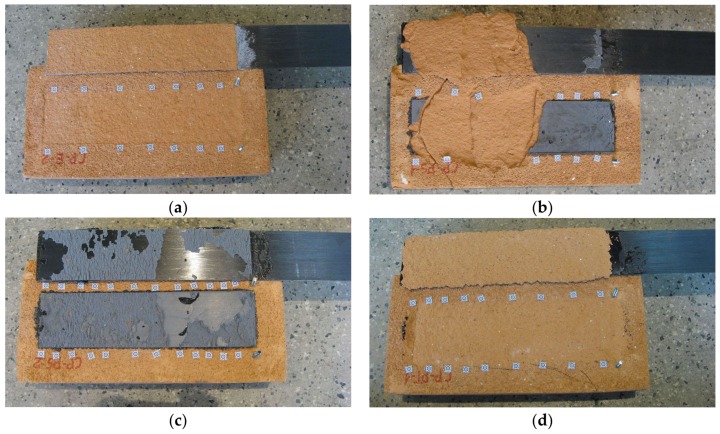
Failure modes observed during the tests of CFRP strips: (**a**) CP-E-2 failure mode type I; (**b**) CP-PS-1 failure mode type I/IV; (**c**) CP-PS-2 failure mode type III/IV; (**d**) CP-PT-1 failure mode type I.

Those specimens with CFRP strips failed mainly due to the debonding of the plate with a cohesive failure within the substrate (type I). Only CP-PS specimens experienced a mixed failure mode—apart from type I failure mode, detachment at the plate-to-adhesive interface (type IV) and debonding at the adhesive (type III) were observed (see Figure 8). Peak loads reached 13.51 kN (CP-E), 17.48 kN (CP-PT), and 22.35 kN (CP-PS), for epoxy-based, polyurethane PT, and polyurethane PS adhesives, respectively.

**Figure 9:**
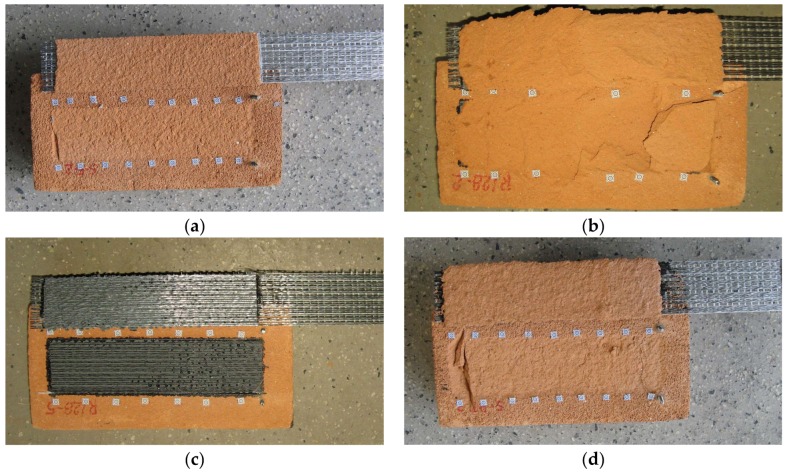
Failure modes observed during the tests of composites reinforced with steel textile S2: (**a**) S2/S-E-2 failure mode type A; (**b**) S2/S-PS-2 failure mode type A; (**c**) S2/S-PS-5 failure mode type C; (**d**) S2/S-PT-3 failure mode type A.

In the case of composites reinforced with steel textile S2 (the second phase of tests), the type of failure mode observed depended strongly on the type of matrix. Specimens S2/S-PS failed due to debonding at the textile-to-matrix interface (type C), whereas specimens S2/S-E and S2/S-PT failed due to a cohesive failure within the substrate (type A) (see Figure 9). The average failure loads were 8.46 kN (S2/S-E), 13.81 kN (S2/S-PT), and 15.16 kN (S2/S-PS) for epoxy-based, polyurethane PT, and polyurethane PS matrices, respectively.

Table 3 shows the experimental results: specimen notation, failure mode, the maximum load F_max_, the calculated average value of the maximum force F_max.av_, corresponding coefficient of variation CoV, the maximum experimental tensile stress in the reinforcement σ_max_, exploitation ratio η. All parameters were computed with reference to the cross-sectional area of the reinforcement. The exploitation ratio η, was defined as σ_max_ over the tensile strength of the related reinforcement (see Table 1).

Typical failure modes obtained in the tests are presented in Figure 8 and Figure 9.

**Figure 10:**
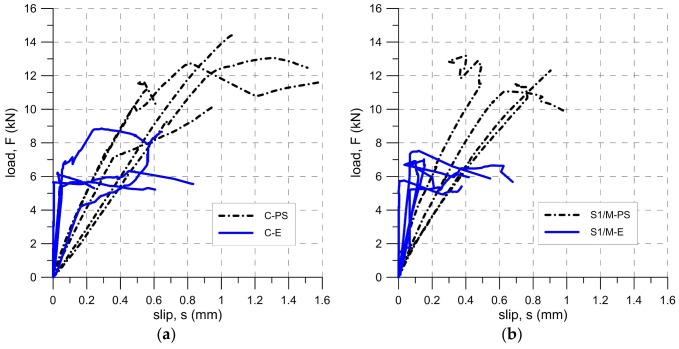
Slip-load curves for TS-1: (**a**) carbon fiber textile and (**b**) steel textile.

**Figure 11:**
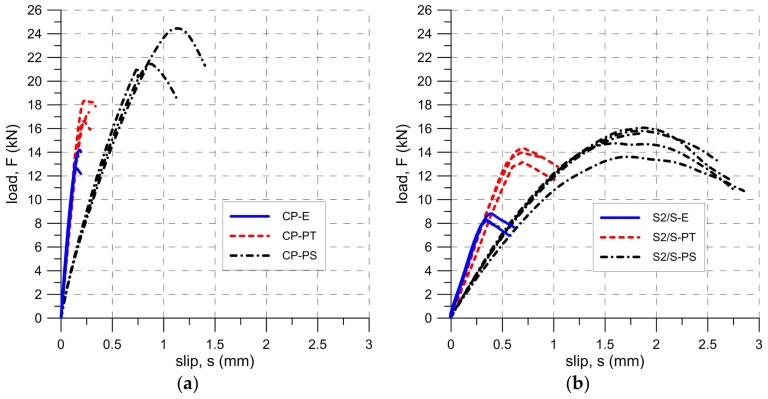
Slip-load curves for TS-2: (**a**) externally bonded CFRP strip and (**b**) steel textile.

The force-slip curves obtained in the tests for TS-1 and TS-2 are presented in Figure 10 and Figure 11. Slips given in Figure 10 (TS-1) were taken from the LVDT located at the loaded end of the brick (transducer LE—see Figure 4b), whereas the slip in the Figure 11 (TS-2) was calculated as an average slip from transducers L1 and L2 (see Figure 5). Visible in Figure 10 (TS-1), instabilities of load-slip curves were caused by a non-stable support of specimens in the C-shaped steel frame. This also could influence a lower load and displacement capacity of the specimens tested in TS-1, in comparison to the same obtained for the specimens tested in TS-2. Another reason of the lower load capacity and displacement capacity of the steel specimens tested in TS-1 (S1/M), in comparison to the same tested in TS-2 (S2/S), could be related to the presence of a glass mesh between the steel fibers and the brick substrate. In the case of S1/M, the glass mesh reduced the bonding area between the adhesive and steel fibers, whereas the lack of this mesh in the contact zone (S2/S) assured full connection between the adhesive and the whole steel reinforcement area. This allowed for a reduction of shear bond stress, and thus, for higher ultimate loads (compare Figure 10b and Figure 11b).

**Figure 12:**
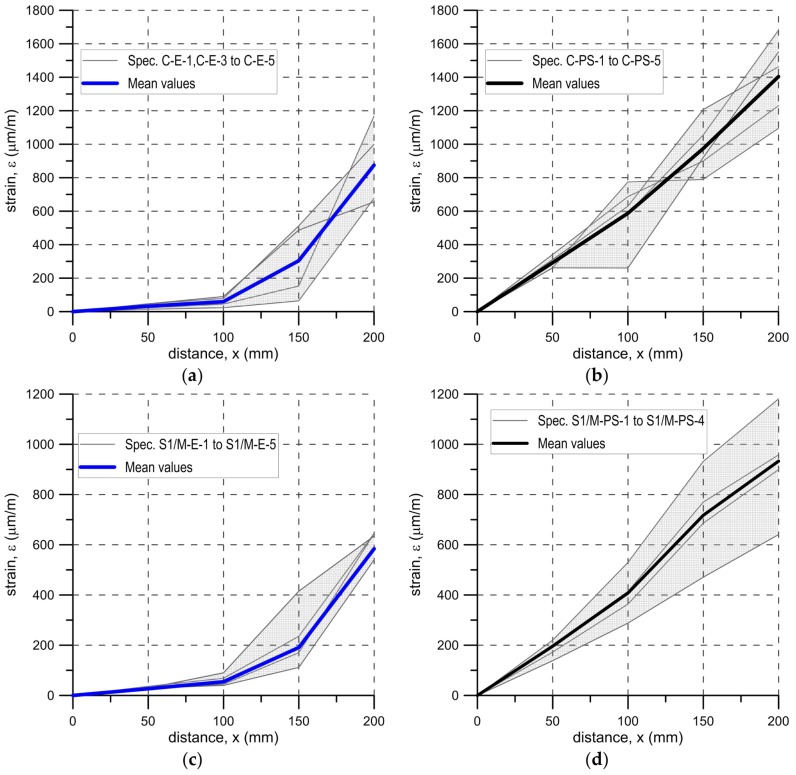
Envelope and mean distribution of strain profiles along bond at F_max_ for tests TS-1: (**a**) C-E specimens; (**b**) C-PS specimens; (**c**) S1/M-E specimens; and (**d**) S1/M-PS specimens.

**Figure 13:**
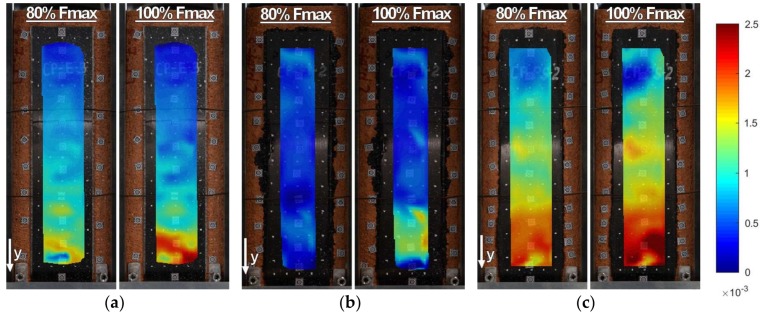
DIC (CivEng-Vision) maps of strains in y direction at 80% F_max_ and 100% F_max_ for specimens: (**a**) CP-E-3; (**b**) CP-PT-2; and (**c**) CP-PS-2.

**Figure 14:**
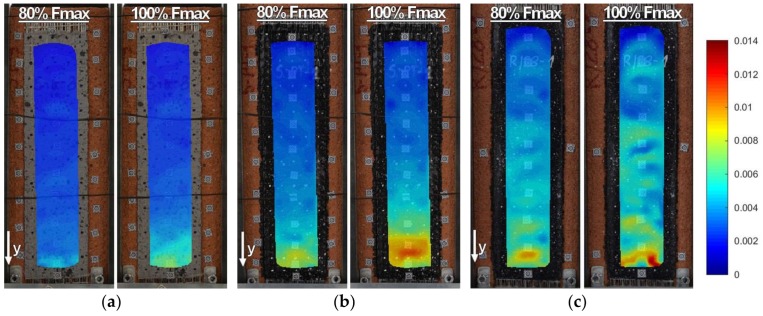
DIC (CivEng-Vision) maps of strains in y direction at 80% F_max_ and 100% F_max_ for specimens: (**a**) S2/S-E; (**b**) S2/S-PT-1; (**c**) S2/S-PS-1.

Strains along the bond length were determined, in order to better understand the bond behavior of the tested strengthening systems. In the case of TS-1 strains, were measured using strain gauges (T0 to T3) applied to the surface of the reinforcement, as shown in Figure 4b. For tests TS-2, strain maps were determined using an optical method, assuming that the DIC method used in the test is reliable enough for this type of specimens [30]. Appropriate strain profiles, obtained from strain gauges for the maximum loads and determined in the center line of bond length (for specimens tested using TS-1), are presented in Figure 12. On the other hand, Figure 13 and Figure 14 presents maps of strains (from DIC), obtained from DIC for 80% and 100% of maximum loads (for specimens tested using TS-2).

A comparison of strain distributions presented for specimens with epoxy and polyurethane PS adhesives indicates that both applied measurement methods (strain gauges and DIC) confirm the occurrence of strain concentrations at the loaded end (Figure 13a and Figure 14a) and on the short effective bonding length (about 100 mm) when stiff epoxy adhesives are used (exponential strain shape—Figure 12a,c). On the other hand, flexible polyurethane adhesives distribute the strains along the bonding length more evenly (triangle strain shape—Figure 12b,d), activating the entire bonding area (Figure 13c and Figure 14c).

## 3. Numerical Analysis

The finite element method (FEM) based numerical analysis of stiff and flexible adhesives was performed to qualitatively compare the results of shear stress distributions and shear strain distributions for bonding materials measured in experiments and computed in numerical simulations of SLST, using Abaqus code.

### 3.1. Material Model Description for Polymer

The hyper-elastic material model for flexible adhesives was based on the Mooney-Rivlin theory in which the strain energy density function (in the stretch λ domain) is given by the equation:

(2) $$W^{M-R}=C_{10}\left(\lambda^{2}+\frac{2}{\lambda }-3\right)+C_{01}\left(\frac{1}{\lambda^{2}}+2\lambda -3\right).$$

The parameters *C*_10_ and *C*_01_ of the hyper-elastic material can be determined by using a uniaxial tension test. For consistency with the linear elasticity theory, in the limit of small strains, the Young’s modulus *E*_0_ and the shear modulus *G*_0_ can be expressed by the parameters *C*_01_ and *C*_10_ as follows:

(3) $$E_{0}=3G_{0}=6\left(C_{10}+C_{01}\right)$$

(4) $$G_{0}=2\left(C_{10}+C_{01}\right).$$

### 3.2. FEM Model Description of SLST

A full 3D model of the SLST specimen was defined in Abaqus 6.14-2 software for the C-PS, S1/M-PS, C-E and S1/M-E specimens (TS-1), respectively, by assuming a symmetrical half of the composite-adhesive-brick assembly. The adhesive layer was modeled with C3D8HR elements and the mechanical behavior of the adhesive layer. In the case of the polyurethane PS, this material was described by the hyper-elastic model with two parameters *C*_01_ = 0.652 MPa and *C*_10_ = 1.211 MPa, after [18]. In the case of the epoxy (Sikadur S330), the material was defined as the isotropic linear elastic (*E* = 4.5 GPa and ν = 0.29). The strips were modeled with C3D8R elements, and the materials were assumed to be isotropic linear elastic materials (*E* = 195 GPa, ν = 0.2 for SRP and *E* = 234 GPa, ν = 0.23 for CFRP). The brick was modeled using C3D8R elements and the isotropic linear elastic material (*E* = 5.76 GPa, ν = 0.2). The load of the SLST specimen was defined, as in the experiment, by the displacement rate of the strip (0.3 mm/min). The analysis for the C-E and S1/M-E specimens was linear, and for the C-PS and S1/M-PS specimens the analysis was fully nonlinear.

**Figure 15:**
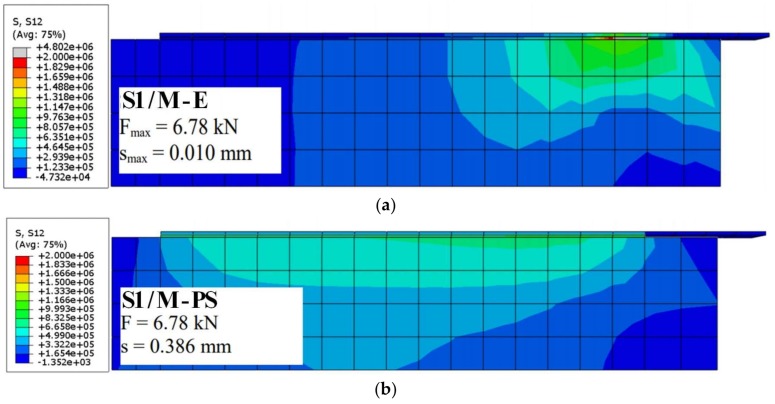

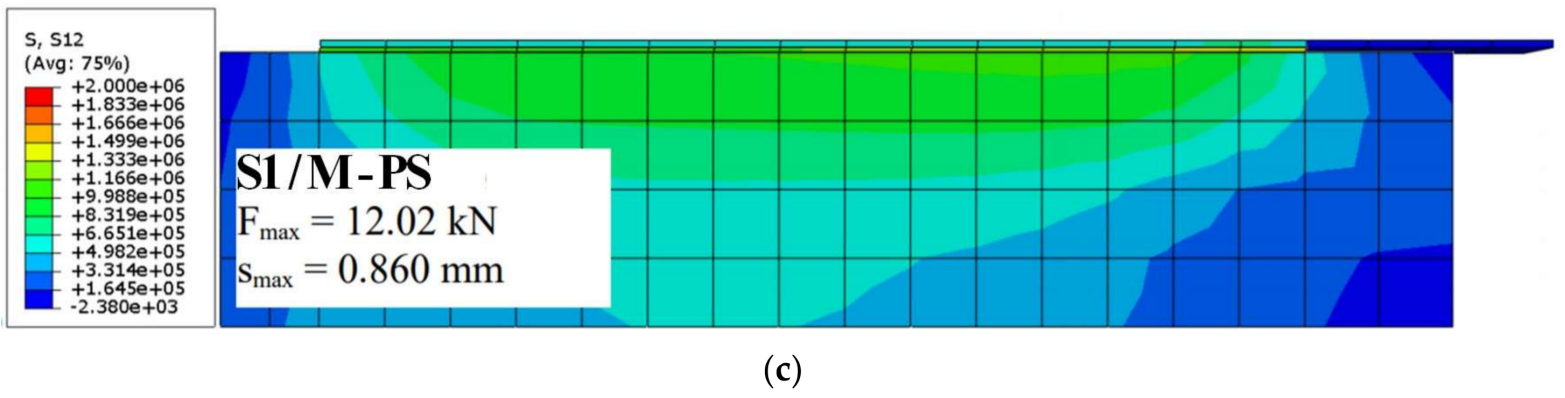
Shear stress distributions computed for: (**a**) S1/M-E with F_max_ for S1/M-E; (**b**) S1/M-PS with F_max_ for S1/M-E; and (**c**) S1/M-PS with F_max_ for S1/M-PS.

The FEM model described above simulates the slip only by assuming a nonzero thickness of the adhesive layer which can be seen in Figure 15 as a layer between the strip on the top and the brick below. The interactions between the strip and the adhesive layer and between the adhesive layer and the brick were assumed to have the form of a tie constraint in Abaqus.

### 3.3. Numerical Results for Shear Stress Distribution

The shear stress maps for S1/M-E and S1/M-PS specimens are presented in Figure 15 (using a similar scale). The presented results confirm that the polyurethane PS adhesive allows for the reduction of high stress concentrations, in comparison to the case of using epoxy adhesive, and is also able to more evenly distribute the shear stress along the bond surface. The shear stress maps for C-E and C-PS are very similar to the previous maps, thus they are not presented here. A comparison of shear stresses was presented for three cases: S1/M-E (epoxy) with F_max_ for S1/M-E (Figure 15a); S1/M-PS (polyurethane PS) with F_max_ for S-E (Figure 15b); and S1/M-PS (polyurethane PS) with F_max_ for S1/M-PS (Figure 15c). This confirms the observations that stiff (epoxy) adhesives cause stress concentrations at the loaded end and activate the short effective bonding length.

### 3.4. Numerical Results for Shear Strain Distribution

**Figure 16:**
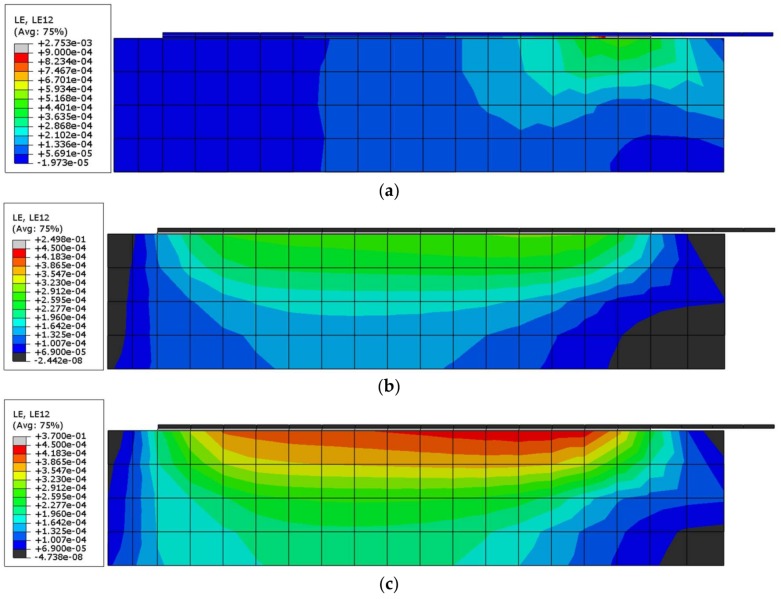
Shear strain distributions computed for: (**a**) S1/M-E with F_max_ for S1/M-E; (**b**) S1/M-PS with F_max_ for S1/M-E; and (**c**) S1/M-PS with F_max_ for S1/M-PS.

The shear strain maps for S1/M-E and S1/M-PS specimens are presented in Figure 16 (using similar scale). The shear strain maps for C-E and C-PS specimens are very similar to the previous maps, thus they are not presented below. The presented results for strain distributions also confirm that the polyurethane PS adhesive allows for the reduction of high strain concentrations, in comparison to the case of using epoxy adhesive, and is also able to more evenly distribute the shear strain along the bond surface.

## 4. Discussion

### 4.1. DIC Ability to Analyse Stiff and Flexible Adhesives in Composite Strengthening

**Figure 17:**
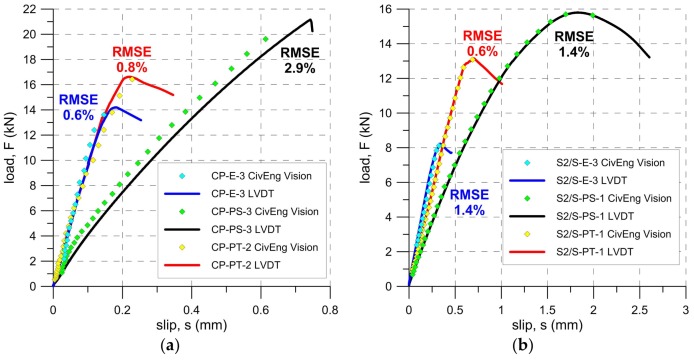
Load-slip response curve provided by LVDT and DIC measurement methods in the shear bond test on: (**a**) CFRP laminates and (**b**) steel fiber textile S2; (RMSE—root mean square errors of LVDT and DIC comparison).

The optical measurement system used in this research is not a commercial solution, but rather an original solution developed at CUT. It was decided that it would be reasonable to compare the results obtained using traditional and optical measurement systems to prove the effectiveness of the second system. The comparison of the results obtained from the LVDTs and DIC method for selected specimens are presented in Figure 17, with root mean square errors (RMSE) computed for the presented data. They varied from 0.6% for S2/S-PT-1 to 2.9% for CP-PS-3, so it can be stated that a good correlation between DIC measurements and LVDT has been observed for both CFRP laminate and steel fiber textile reinforced composites. Thus, further calculations using an optical measurement system have been carried out to expand the set of results originally received using LVDTs and to generate strain fields on the specimen surface, as is described below.

DIC allows for the presenting of maps of strains calculated for the entire bonded area. The strain maps in the vertical direction, calculated for the load levels of 80% and 100% F_max_ (Figure 13 and Figure 14), allow for the observing of uneven changes in strain distributions (making failure process more understandable), which are unable to be noticed using traditional measurement devices. DIC confirms the observations obtained from strain gauges (see results for TS-1) that an effective transfer length of composites with flexible adhesive/matrix is longer than the bonding length of strengthening systems with a stiff epoxy adhesive/matrix.

On the other hand, DIC records displacements only on the visible surface of the specimen, so no more direct information is provided on the textile embedded within the matrix for wet lay-up strengthening systems. It should be noted that only if there is a full bond between the reinforcement and matrix strains measured at the composite surface can be treated as strains of the entire composite. Only in the case of FRP laminates, which are loaded in their plane, strains recorded using DIC can be considered as strains of the composite.

### 4.2. Differences in Work of Brick Specimens Strengthened with Composites Bonded on Stiff and Flexible Adhesives

**Figure 18:**
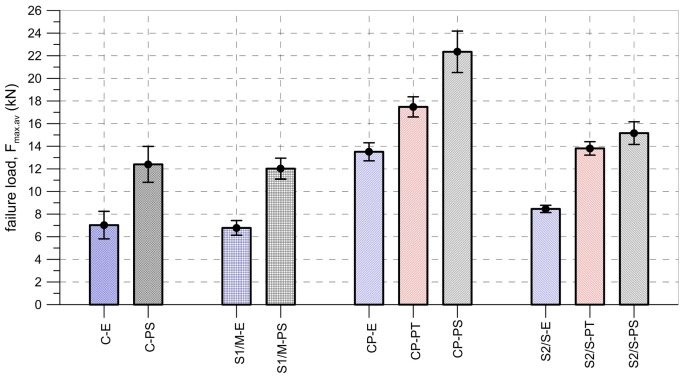
Average failure loads obtained in tests presented with their standard deviations.

Figure 18 shows a comparison of the average failure loads obtained in the tests performed on different strengthening systems. Regardless of the type of strengthening system, higher failure loads were observed for those specimens made with flexible adhesives/matrices. The application of flexible adhesives/matrices allowed for an increase in strengthening-to-brick bond capacity compared to stiff (epoxy) ones. The differences in failure loads for flexible and stiff adhesives/matrices varied between 65% for CFRP laminate and 79% for steel fiber textile S2/S.

**Figure 19:**
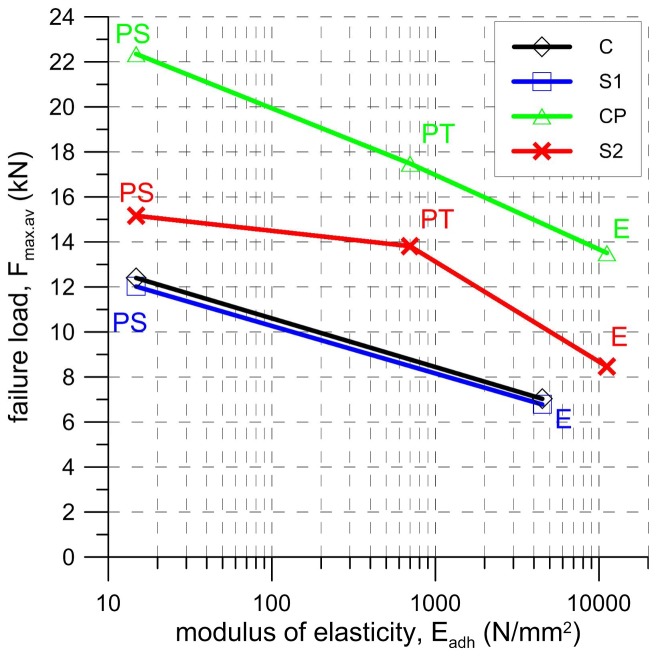
Average failure load (F_max,av_) versus modulus of elasticity of adopted adhesive (E_adh_) for various textiles/laminates (C-carbon textile, S1, S2—steel textiles, CP—CFRP strip).

As can be seen in Figure 10 and Figure 11, both the slope of force-slip curves in the first (linear) phase of tests and the slip values at failure load depend on the modulus of elasticity of the material of matrix/adhesive. The stiffer the material used, the smaller the slip at the failure load and a steeper slope of force-slip curve has been observed. The modulus of elasticity of the adhesive/matrix material also influenced the failure load. As is presented in Figure 19, regardless of adopted strengthening system, the failure load increased with a decrease in the stiffness of matrix/adhesive. It should be noted here that the bond strength depends not only on the mechanical parameters of the adhesive, but also on the mechanical parameters of the substrate. The research presented here was conducted only on one type of substrate—clay brick. This type of substrate was chosen because these bricks are characterized by low coefficients of variation in their strength parameters [6]. When structural strengthening of a historical structure is considered, the large variance of brick strength, even within one masonry wall, should be taken into consideration [48]. In particular, designers should be aware that bond strength is also conditioned by the conditions of the bricks, and it strongly depends on environmental factors like moisture and salt crystallization, which can occur in historical buildings. These phenomena determine not only the brick’s mechanical degradation [49], but also the bond strength of the composite strengthening system [19]. The influence of moisture and salt crystallization should be taken into account in future research, especially on the FRPU strengthening system.

Additionally, the failure loads presented in this paper were obtained from short-term (quasi-static loading) single lap shear tests. It should be noted that after an application of the strengthening on an existing structural element, the strengthening will be under dead and live loads, so long-term effects occur. In this case, the influence of slow crack propagation, which can cause deboning after some years, should be considered [50,51]. 

**Figure 20:**
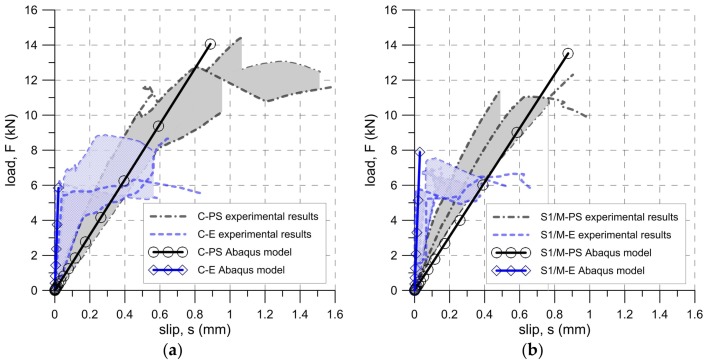
Comparison of load-slip curves from experimental tests (TS-1) and numerical analysis: (**a**) carbon fiber reinforced composite C and (**b**) steel fiber reinforced composite S1.

The difference in stiffness was also taken into consideration in the numerical analysis when models parameters were matched. Figure 20 illustrates a good agreement in stiffness between the load-slip curves, determined both experimentally and numerically.

**Figure 21:**
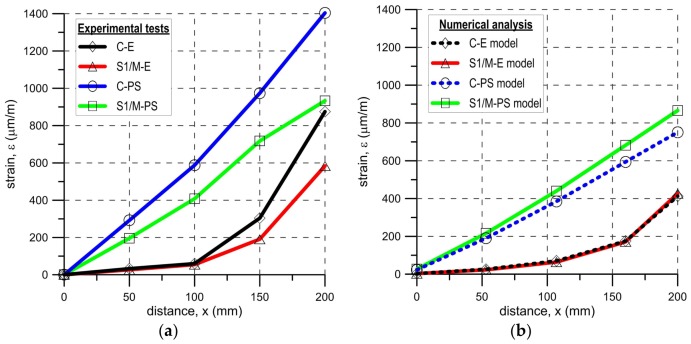
Typical mean strain profiles along bonds at F_max_: (**a**) experimental tests TS-1 and (**b**) numerical analysis.

During the tests conducted using test set-up TS-1, the axial strain of the composite along its bonded length was measured and recorded for all tested specimens at the maximum load. The average strain profiles are presented in Figure 21a. In the case of the specimens with epoxy resin reinforced with carbon and steel fabrics, the curves are exponential with an effective transfer length of about 100 mm. The strain profiles for the polyurethane matrix are almost linear and the required bond length is likely longer than the adopted one. A similar strain distribution was obtained in the numerical analysis (Figure 21b). Small differences are observed, in comparison to the experimental ones (Figure 21a), caused by inaccuracies of the numerical model.

**Figure 22:**
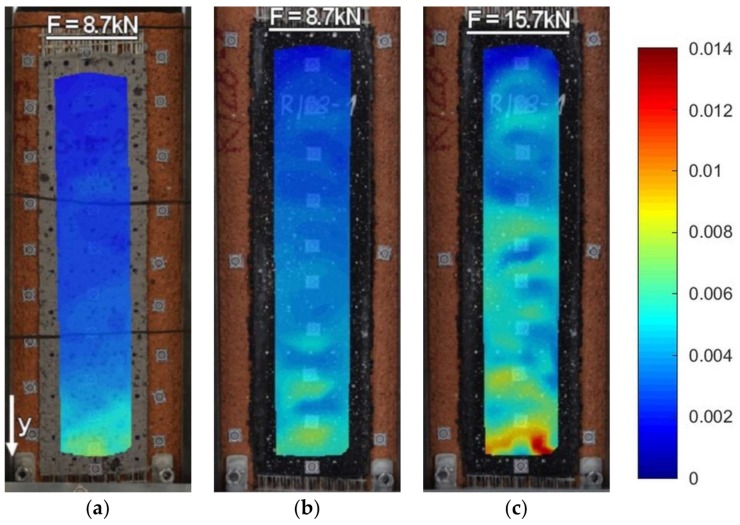
DIC (CivEng-Vision) maps of strains for specimens: (**a**) S2/S-E-3 at a load F_max_(S2/S-E-3); (**b**) S2/S-PS-1 at a load F_max_(S2/S-E-3); (**c**) S2/S-PS-1 at a load F_max_(S2/S-PS-1).

**Figure 23:**
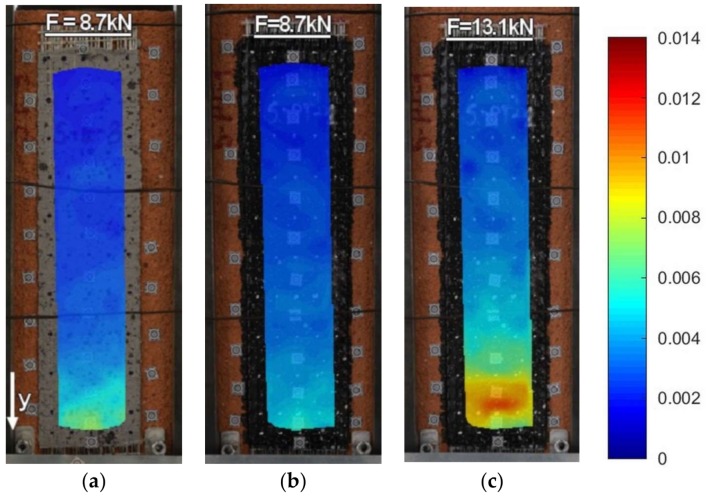
DIC (CivEng-Vision) maps of strains for specimens: (**a**) S2/S-E-3 at a load F_max_(S2/S-E-3); (**b**) S2/S-PT-1 at a load F_max_(S2/S-E-3); (**c**) S2/S-PT-1 at a load F_max_ (S2/S-PT-1).

**Figure 24:**
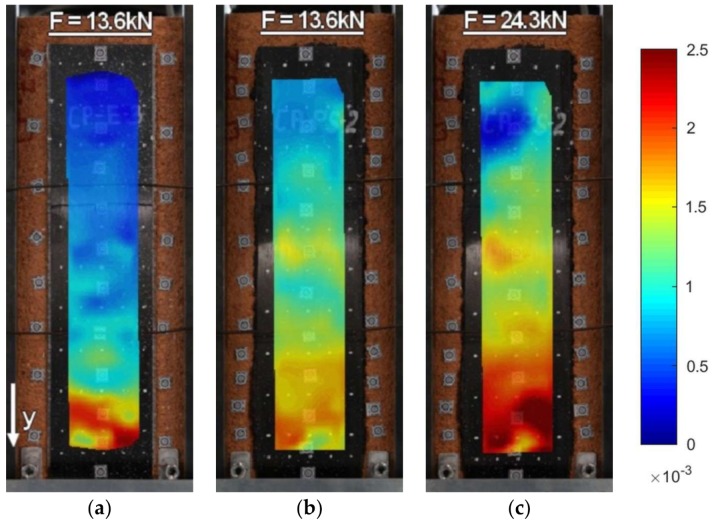
DIC (CivEng-Vision) maps of strains for specimens: (**a**) CP-E-3 at a load F_max_(CP-E-3); (**b**) CP-PS-2 at a load F_max_(CP-E-3); (**c**) CP-PS-2 at a load F_max_(CP-PS-2).

**Figure 25:**
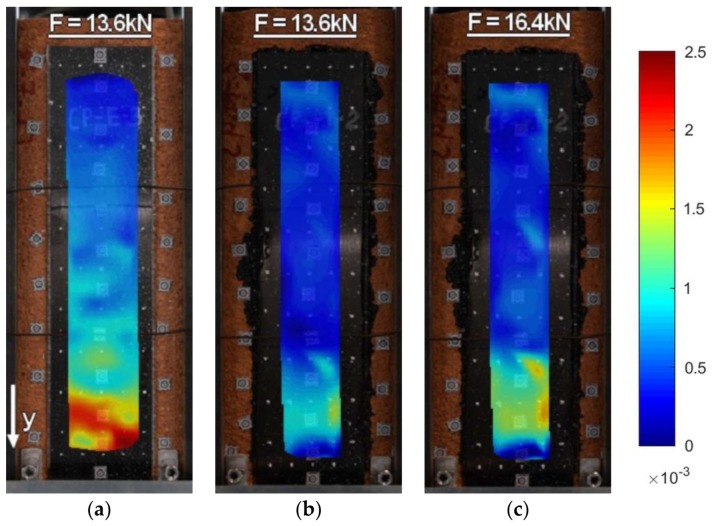
DIC (CivEng-Vision) maps of strains for specimens: (**a**) CP-E-3 at a load F_max_(CP-E-3); (**b**) CP-PT-2 at a load F_max_(CP-E-3); (**c**) CP-PT-2 at a load F_max_(CP-PT-2).

A discussion on the strain distribution along the bond length can be had, based on the strain maps obtained using the DIC method. In figures “a” of Figure 22, Figure 23, Figure 24 and Figure 25 the strain for a stiff epoxy adhesive/matrix at its failure load is presented, while in Figure 22b, Figure 23b, Figure 24b, Figure 25b, the strain for flexible polyurethane adhesive/matrix at the load equal to failure load of epoxy ones is shown. So, these figures (“a” and “b”) provide information on how the load applied to the reinforcement is transferred to the brick. It can be noticed that, in the case of epoxy, a shear stress between the strengthening and substrate concentration occurred close to the loaded end, whereas the flexible PS allowed for a more uniform distribution of shear stresses. Using a flexible polyurethane PS matrix causes the effective bond length to be longer than it is for the epoxy one. This phenomenon is clearly visible in the case of the CFRP strips, for which the strain maps are presented in Figure 24a,b. The conclusions made using the DIC method are similar to those observations obtained from numerical analysis (see Figure 16). Additionally, the strain distributions for the specimens with polyurethane matrix/adhesive at their failure load are presented in Figure 22c, Figure 23c, Figure 24c, Figure 25c.

## 5. Conclusions

In the calculations of FRP/SRP and FRCM/SRC (TRM/SRM), the influence of adhesive properties is neglected (an assumption of perfect contact between the composite and substrate is considered in guidelines, e.g., ACI 440.2R-08, CNR-DT 200/2004). The results presented in this paper indicate that properties of adhesives in composite-to-substrate strengthening systems should be taken into consideration. This is the case, especially, when flexible adhesives (made from special polyurethanes—i.e., PS and PT) reduce the shear stress concentrations and redistribute them to the larger bond area, making the composite strengthening system of a higher load capacity. This phenomenon was confirmed in this paper, as well as in other papers that present tests results made on FRPU/SRPU systems in various European laboratories, by experimental and numerical analysis (with flexible adhesives modeled using the hyper-elastic model). The analysis confirmed that composite-to-brick strengthening systems with flexible adhesives are more effective on brittle substrates than stiff ones, since the flexible behavior of adhesives is relevant and should be taken into consideration in analysis and calculations.

The use of flexible adhesives in composite-to-substrate strengthening systems creates new opportunities for practical applications of composite materials, when adhesive properties are taken into consideration in calculations and experimental investigations. This is a novel approach towards the implementation of composite systems, which will require confirmation in future research.

The optical measurement allows for the obtaining of better results on the point grid without increasing the number of sensors; however, this information is not directly accessible and requires processing of the collected material, contrary to conventional measurements in which the measured value is available directly from the sensor. It should also be noted that an optical measurement can be performed in specific lighting conditions. A stable base for the components of the vision system must also be ensured.

When structural strengthening of a historical structure is considered, additional phenomena, which were not analyzed in this paper, such as environmental factors, variability of mechanical properties of materials in existing masonry structures, long time behavior, etc. must be taken into consideration.

## Figures and Tables

**Table 1:** Mechanical properties of the materials used in the tests.

Material/Mechanical Property	Value
**Clay Brick ^1.^**	
compressive strength	19.8 N/mm^2^
flexural strength	3.66 N/mm^2^
splitting tensile strength	2.46 N/mm^2^
Young’s modulus	5760 N/mm^2^
**Epoxy adhesive/Sikadur 330/ ^2^**	
tensile strength	30 N/mm^2^
Young’s modulus	4500 N/mm^2^
strain at peak load	0.90%
**Epoxy adhesive/Sikadur 30 Normal/ ^2^**	
tensile strength	26 N/mm^2^
Young’s modulus	11,200 N/mm^2^
**Polyurethane adhesive/PS/ ^2^**	
tensile strength	2.5 N/mm^2^
Young’s modulus	16 N/mm^2^
strain at peak load	45%
**Polyurethane adhesive/PT/ ^2^**	
tensile strength	20 N/mm^2^
Young’s modulus	700 N/mm^2^
strain at peak load	10%
**Carbon textile/FIDCARBON UNI 320 HT240/ ^1^**	
tensile strength	2735 N/mm^2^
Young’s modulus	234,000 N/mm^2^
nominal thickness	0.170 mm
**CFRP plate/Sika CarboDur S1012/ ^2^**	
tensile strength	2900 N/mm^2^
Young’s modulus	165,000 N/mm^2^
plate thickness	1.2 mm
**Steel textile S1/FIDSTEEL 3X2-B 12-12-500/ ^1^**	
tensile strength	2997 N/mm^2^
Young’s modulus	195,000 N/mm^2^
nominal thickness	0.231 mm
**Steel textile S2/Kerakoll GeoSteel G2000/ ^3^**	
tensile strength	3083 N/mm^2^
Young’s modulus	183,000 N/mm^2^
nominal thickness	0.254 mm

^1^ According to [6], ^2^ specified by manufacturer, ^3^ according to [44].

**Table 2:** Tested specimens.

Notation	No. of Specimens	Description of Strengthening System	Test Set-Up
C-E	5	**carbon fiber textile** embedded in epoxy based matrix Sikadur 330	TS-1
C-PS	5	**carbon fiber textile** embedded in polyurethane PS matrix	TS-1
S1/M-E	5	**steel fiber textile S1** embedded in epoxy based matrix Sikadur 330—glass mesh side towards the substrate	TS-1
S1/M-PS	4	**steel fiber textile S1** embedded polyurethane PS matrix—glass mesh side towards the substrate	TS-1
CP-E	3	**pultruded CFRP laminate** bonded using epoxy based adhesive Sikadur 30	TS-2
CP-PS	3	**pultruded CFRP laminate** bonded using polyurethane PS adhesive	TS-2
CP-PT	3	**pultruded CFRP laminate** bonded using polyurethane PT adhesive	TS-2
S2/S-E	3	**steel fiber textile S2** embedded in epoxy based matrix Sikadur 30—steel cords side towards the substrate	TS-2
S2/S-PS	3	**steel fiber textile S2** embedded polyurethane PS matrix—steel cords side towards the substrate	TS-2
S2/S-PT	3	**steel fiber textile S2** embedded polyurethane PT matrix—steel cords side towards the substrate	TS-2

**Table 3:** Experimental results of bond tests.

Type of Composite	Specimen	Failure Mode	F_max_ (kN)	F_max.av_ (kN)	CoV (%)	σ_max_ (MPa)	η (-)
CFRP	C-E-1	A	6.32				
	C-E-2	A	7.71				
	C-E-3	A	8.85	7.03	17.3	852	0.31
	C-E-4	A	6.05				
	C-E-5	A	6.23				
CFRPU	C-PS-1	- ^1^	12.73				
	C-PS-2	- ^1^	14.40				
	C-PS-3	C	11.64	12.40	12.8	1503	0.55
	C-PS-4	A	10.18				
	C-PS-5	A	13.05				
SRP	S1/M-E-1	A	6.66				
	S1/M-E-2	A	5.76				
	S1/M-E-3	A	6.91	6.78	9.6	597	0.20
	S1/M-E-4	A	7.53				
	S1/M-E-5	A	7.02				
SRPU	S1/M-PS-1	C	11.06				
	S1/M-PS-2	A	12.31	12.02	7.7	1059	0.35
	S1/M-PS-3	A	13.18				
	S1/M-PS-4	A	11.53				
CFRP	CP-E-1	I	13.69				
	CP-E-2	I	12.64	13.51	5.9	135	0.05
	CP-E-3	I	14.20				
CFRPU	CP-PS-1	I/IV	21.44				
	CP-PS-2	III/IV	24.45	22.35	8.2	224	0.08
	CP-PS-3	I	21.16				
CFRPU	CP-PT-1	I	17.42				
	CP-PT-2	I	16.62	17.48	5.1	175	0.06
	CP-PT-3	I	18.39				
SRP	S2/S-E-1	A	8.83				
	S2/S-E-2	A	8.32	8.46	3.8	655	0.21
	S2/S-E-3	A	8.23				
SRPU	S2/S-PS-1	C(D)	15.80				
	S2/S-PS-2	A	15.58				
	S2/S-PS-3	C	16.07	15.16	6.6	1174	0.38
	S2/S-PS-4	C	14.75				
	S2/S-PS-5	C	13.61				
SRPU	S2/S-PT-1	A	13.15				
	S2/S-PT-2	A	14.31	13.81	4.3	1070	0.35
	S2/S-PT-3	A	13.96				

^1^ Non-standard failure mode due to shear failure of the brick.
